# Application of DKI and IVIM imaging in evaluating histologic grades and clinical stages of clear cell renal cell carcinoma

**DOI:** 10.3389/fonc.2023.1203922

**Published:** 2023-10-26

**Authors:** QiChao Cheng, AnLi Ren, XingHua Xu, Zhao Meng, Xue Feng, Dmytro Pylypenko, WeiQiang Dou, DeXin Yu

**Affiliations:** ^1^ Department of Radiology, Qilu Hospital of Shandong University, Jinan, China; ^2^ Department of Radiology, Affiliated Hospital of Shandong University of Traditional Chinese Medicine, Jinan, China; ^3^ MR Research, GE Healthcare, Beijing, China

**Keywords:** diffusion kurtosis imaging, intravoxel incoherent motion, histologic grades, clinical stages, clear cell renal cell carcinoma

## Abstract

**Purpose:**

To evaluate the value of quantitative parameters derived from diffusion kurtosis imaging (DKI) and intravoxel incoherent motion (IVIM) in differentiating histologic grades and clinical stages of clear cell renal cell carcinoma (ccRCC).

**Materials and methods:**

A total of 65 patients who were surgically and pathologically diagnosed as ccRCC were recruited in this study. In addition to routine renal magnetic resonance imaging examination, all patients underwent preoperative IVIM and DKI. The corresponding diffusion coefficient (D), pseudo-diffusion coefficient (D*), perfusion fraction (f), mean diffusivity (MD), kurtosis anisotropy (KA), and mean kurtosis (MK) values were obtained. Independent-samples t-test or Mann–Whitney U test was used for comparing the differences in IVIM and DKI parameters among different histologic grades and clinical stages. The diagnostic efficacy of IVIM and DKI parameters was evaluated using the receiver operating characteristic (ROC) curve. Spearman’s correlation analysis was used to separately analyze the correlation of each parameter with histologic grades and stages of ccRCC.

**Results:**

The D and MD values were significantly higher in low-grade ccRCC than high-grade ccRCC (all *p* < 0.001) and in low-stage than high-stage ccRCC (all *p* < 0.05), and the f value of high-stage ccRCC was lower than that of low-stage ccRCC (*p* = 0.007). The KA and MK values were significantly higher in low-grade than high-grade ccRCC (*p* = 0.000 and 0.000, respectively) and in low-stage than high-stage ccRCC (*p* = 0.000 and 0.000, respectively). The area under the curve (AUC) values of D, D*, f, MD, KA, MK, DKI, and IVIM+DKI values were 0.825, 0.598, 0.626, 0.792, 0.750, 0.754, 0.803, and 0.857, respectively, in grading ccRCC and 0.837, 0.719, 0.710, 0.787, 0.796, 0.784, 0.864, 0.823, and 0.916, respectively, in staging ccRCC. The AUC of IVIM was 0.913 in staging ccRCC. The D, D*, and MD values were negatively correlated with the histologic grades and clinical stages (all *p* < 0.05), and the KA and MK values showed a positive correlation with histologic grades and clinical stages (all *p* < 0.05). The f value was also negatively correlated with the ccRCC clinical stage (*p* = 0.008).

**Conclusion:**

Both the IVIM and DKI values can be used preoperatively to predict the degree of histologic grades and stages in ccRCC, and the D and MD values have better diagnostic performance in the grading and staging. Also, further slightly enhanced diagnostic efficacy was observed in the model with combined IVIM and DKI parameters.

## Introduction

Renal cell carcinoma (RCC) is a common malignancy in the urologic system, with a 2%–3% annual increase worldwide (1). Clear cell RCC (ccRCC) is the most common type of RCC. With the advancement of imaging techniques, such as ultrasound (US), computed tomography (CT), magnetic resonance imaging (MRI), and PET-CT/MRI, small and low-stage kidney tumors can now be easily detected (2). While radical nephrectomy was once considered the standard curative therapy, partial nephrectomy is now preferred due to its better preservation of renal function (3). Relevant studies have confirmed that partial nephrectomy is widely employed for localized renal tumors. The study, as evidenced by the references you provided, demonstrates that the glomerular filtration rate, chronic kidney disease prevalence, and operative time in the off-clamp partial nephrectomy group were superior to those in the on-clamp group (4, 5). Studies have shown that the histologic differentiation of RCC is an important prognostic factor for patients undergoing partial nephrectomy. High-grade RCC is more aggressive and is associated with a higher risk of relapse or metastasis after surgery (3, 6). Therefore, preoperative prediction of the histologic grade and clinical stage of ccRCC is essential for the development of effective therapeutic strategies.

Compared with US and CT examination, MRI has good spatial and contrast resolution without ionizing radiation burden. Therefore, it was considered a proper method for preoperative assessment for the prognosis of ccRCC (7). MRI should be carried out by contrast injection in detecting malignant lesions; although gadolinium contrast is safer, patients with chronic renal were also affected by the use of gadolinium contrast material (8, 9). Diffusion-weighted imaging (DWI) was mainly applied to quantify the diffusion of water molecules and provided information on cellular density, membrane integrity, and tissue perfusion, which can distinguish viable from necrotic tumors in a non-invasive manner (10). Several previous studies on DWI have demonstrated good value in the grades and stages of renal tumors and differential diagnosis between renal benign and malignant tumors (11–13). However, due to the complexity and restriction of microstructures and water molecule diffusion, DWI is primarily used to quantify the diffusion of water molecules with a Gaussian distribution and cannot accurately reflect the information of the lesion. Moreover, the original apparent diffusion coefficient (ADC) values do not distinguish between the pure diffusive motion of water molecules and the effects of microcapillary perfusion (14, 15). In recent years, with the development of MRI, more advanced intravoxel incoherent motion (IVIM) and diffusion kurtosis imaging (DKI) models have been developed on the basis of DWI. By using multiple b values, IVIM may help to evaluate tissue microcapillary perfusion and provide an accurate characterization of tissue diffusivity motion (16). A previous study has shown that IVIM is helpful for distinguishing RCC from fat-poor angiomyolipoma (17). In addition, IVIM-derived parameters showed important value in the assessment of different renal tumor subtypes (18). DKI is an advanced DWI model that reflects tissue complexity by using higher b values and quantifies the non-Gaussian behavior of diffusion and the excess kurtosis of tissue (16, 19). Compared with the DWI model, the DKI model is mainly applied to detect non-Gaussian water molecule motion to reflect the lesion microstructure and identify tumor and necrotic tissue (20). Previous studies have shown that DKI is able to distinguish different types of RCCs (21, 22). Meanwhile, related studies have revealed that different DKI parameters have certain values in ccRCC grade (23, 24). Based on the different advantages of IVIM and DKI, the different models could show different features of ccRCC tissue, and it may be valuable to explore grading and staging of the ccRCC. Yang L et al. (14) showed that IVIM and DKI were helpful in the assessment of tumor staging and grading after neoadjuvant chemoradiotherapy in patients with locally advanced rectal cancer. However, in ccRCC-related results, previous studies were limited to IVIM or DKI for ccRCC grading or staging; no studies may have applied both IVIM and DKI techniques in ccRCC systematic histologic grades and clinical stages diagnosis.

Therefore, the purpose of our study was to investigate the value of IVIM and DKI parameters in differentiating ccRCC histologic grades and clinical stages, which was further helpful for the management of therapeutic strategies.

## Materials and methods

### Patients

**Figure 1:**
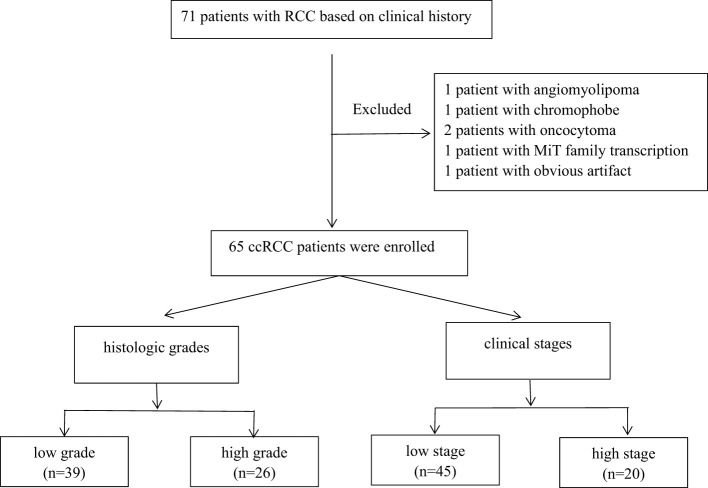
The flowchart of ccRCC. ccRCC, clear cell renal cell carcinoma.

This retrospective study was approved by the Qilu Hospital of Shandong University ethics committee, and written informed consent was provided by all the patients. A total of 71 patients with RCC based on clinical history from September 2022 to January 2023 were recruited. Postoperative pathologies of five patients showed other renal tumors (angiomyolipoma = 1, chromophobe = 1, oncocytoma = 2, and MiT family transcription = 1), and one patient with obvious image artifact was excluded. Finally, 65 ccRCC patients were enrolled in this study, including 43 men and 22 women. The age range was between 27 and 76 years, and the average age was 54 years. The tumor types were classified into four grades regarding the tumor nuclear size, shape, and chromatin pattern as described by the World Health Organization/International Society of Urological Pathology (WHO/ISUP) grading system. After the cytological assessment, the tumors were merged into two groups: low-grade (WHO/ISUP grades 1 and 2, n = 39) and high-grade (WHO/ISUP grades 3 and 4, n = 26) RCCs. The tumor node metastasis (TNM) classification of ccRCC was in four stages and then merged into two groups: low-stage (stages 1 and 2, n = 45) and high-stage (stages 3 and 4, n = 20) RCC. **Figure 1** shows the flowchart of ccRCC.

### MRI acquisition

All subjects underwent a 3.0T MRI scan (Discovery 750w, GE Healthcare, Chicago, IL, USA), with a 24-channel abdominal phased array coil, in a supine and foot-first position. Pre-inspection preparation was performed including fasting over 4 hours and breath-holding practice. MRI scan sequences included axial T1-weighted imaging (T1WI), fat-saturated T2-weighted imaging (T2WI), and dynamic contrast-enhanced MRI (DCE-MRI). IVIM and DKI imaging were performed on the kidney after acquiring the corresponding T2-weighted anatomical images. For IVIM, a single-shot spin-echo echo-planar imaging sequence was applied in axial view with 10 b values (25, 50, 75, 100, 150, 200, 400, 600, 800, and 1,000 s/mm^2^) and respiratory triggered: repetition time/echo time (TR/TE) = 4,000/80.4 ms; field of view (FOV) = 36 × 36 cm^2^; matrix = 128 × 128; slice thickness = 5 mm; scan time = 276 s. For DKI, a separate respiratory-triggered single-shot spin-echo echo-planar imaging sequence with 3 b values (0, 500, and 1,000 s/mm^2^) and 15 diffusion directions per b value was applied. Other scan parameters were as follows: TR/TE = 6,000/126 ms; FOV = 36 × 36 cm^2^; matrix = 128 × 128; slice thickness = 4 mm; scan time = 128 s.

### Data post-processing

All IVIM and DKI data were post-processed with vendor-provided analytic software embedded in the GE ADW4.6 workstation. The resulting IVIM- and DKI-derived parametric maps were generated. The IVIM model was Sb/S0 = (1 − f).exp (−bD) + f.exp (−b [D* + D]) (25), where D is the pure molecular diffusion coefficient, D* is the pseudo-diffusion coefficient representing incoherent microcirculation of the capillary networks, and f is the perfusion fraction. The DKI model was Sb/S0 = exp (−b MD + b2·MD2·MK/6), where MK is the mean kurtosis (MK), and mean diffusion (MD) value is similar to the corrected average ADC value (26). Meanwhile, the kurtosis anisotropy (KA) value was calculated with the DKI model.

The region of interest (ROI) was selected and drawn separately by two abdominal radiologists with over 10 years of experience in MRI protocols. The size and location of the ROI were consistent on the IVIM and DKI parameter maps. The reviewers were blinded to the provided clinical data and pathological diagnosis. The criteria for ROI selection included combining conventional T2WI images, setting the large lesion dimension as the ROI on the b0 image and co-registering other b values of IVIM and DKI images to the b0 image. The scope of the lesion was made as large as possible, and internal areas with necrosis, calcification, and bleeding were excluded. Clear cell RCCs are classically T2 hyperintense, and hemorrhage can be variable on T2WI alone (21).

### Histologic grades

The surgically resected kidney specimens were used for the pathological evaluation and assessed by a urological pathologist who has 12 years of experience and was blinded to clinical data. Histologic grade was classified according to the criteria of the WHO/ISUP grade: grade 1, the nucleolus was absent or not obvious at ×400 magnification; grade 2, under the microscope at ×400 magnification, the tumor cells showed clear nucleoli, but at ×100, the nucleoli were not obvious or unclear; grade 3, the nucleoli were clear at ×100 magnification; grade 4, tumor giant cells, sarcomatoid differentiation, and/or rhabdoid morphology (27). The tumors were merged into two groups: low-grade (WHO/ISUP grades 1 and 2) and high-grade (WHO/ISUP grades 3 and 4) ccRCC (28).

### Clinical stages

Clinical stages were classified according to the criteria of the TNM classification based on the American Joint Committee on Cancer (29): stage 1, the ccRCC tumor was confined to the renal parenchyma, and the maximum diameter of the mass was less than 7 cm; stage 2, the tumor was confined to the renal parenchyma, and the maximum diameter of the tumor was >7 cm; stage 3 refers to tumor thrombus in the renal vein or its branches, upper and lower vena cava, or tumor invasion of the renal pelvis and calyces, perirenal or sinus fat but not beyond the renal fascia; stage 4, the tumor broke through the renal fascia. The tumors were merged into two groups: low-stage (stages 1 and 2) and high-stage (stages 3 and 4) RCC.

### Statistical analysis

Statistical analyses were performed using the SPSS 22 software package (IBM, Armonk, NY, USA). According to the characteristics of data distribution, quantitative data were expressed as the mean ± standard deviation. Inter-class correlation coefficient (ICC) analysis was used to evaluate the interobserver agreement of IVIM and DKI parameter measurements (ICC 0.61–0.80 indicates good, and >0.8 indicates excellent). Independent-samples t-test and Mann–Whitney U test were used to evaluate the differences in IVIM and DKI parameters between high-grade and low-grade as well as between high-stage and low-stage ccRCC. Parameters with *p* < 0.05 were selected for further multivariate logistic regression (30, 31). Receiver operating characteristic (ROC) analysis was performed using MedCalc software (version 11.4.2.0, Ostend, Belgium) to obtain the area under the curve (AUC), sensitivity, and specificity of low and high grades and stages of ccRCC for different parameters and their combinations; optimal cutoff points of different parameters were also determined. Spearman’s correlation analysis was used to analyze the correlation between each IVIM and DKI parameter and the histologic grade and clinical stage of ccRCC. The differences were considered statistically significant at *p* < 0.05.

## Results

### Clinical data

**Figure 2:**
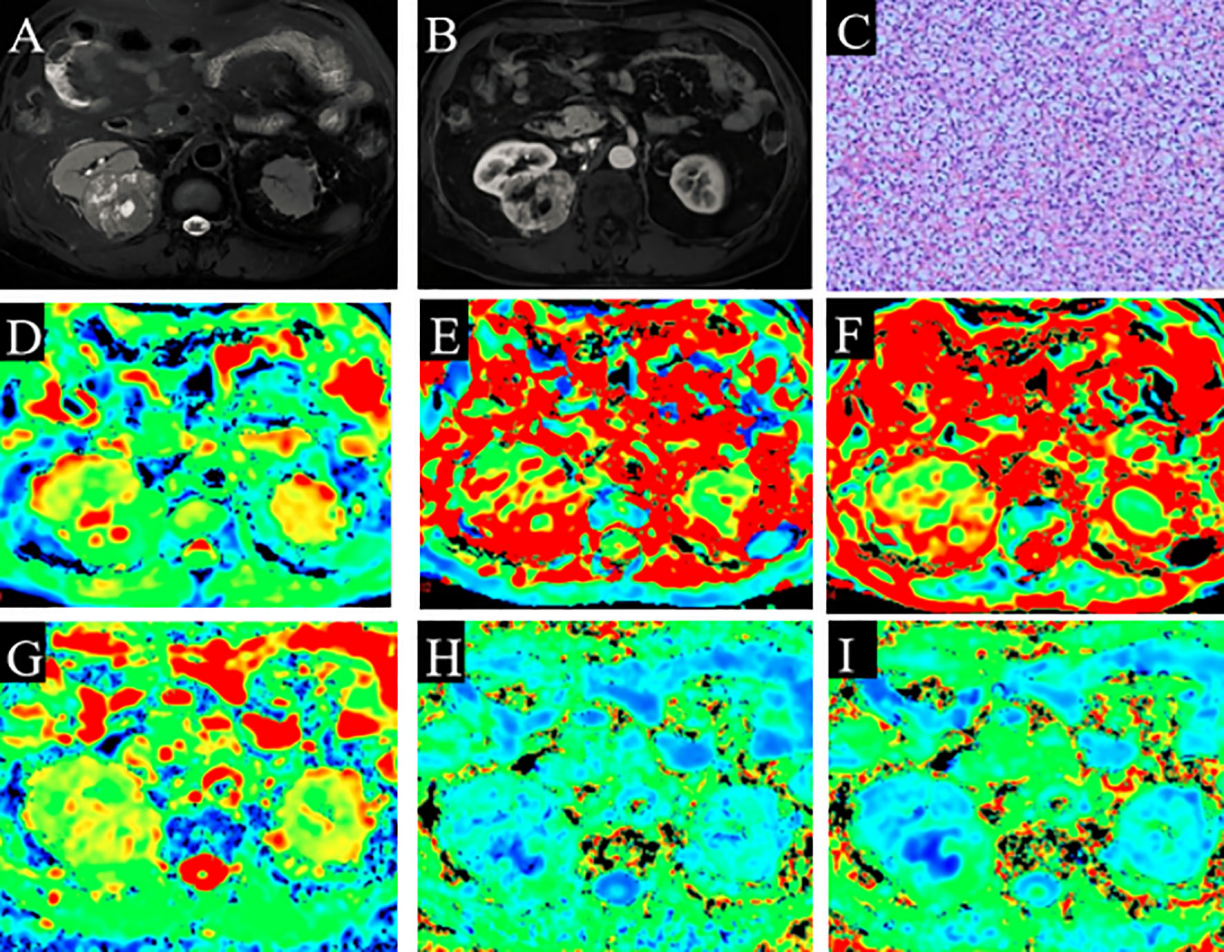
A 60-year-old man with ccRCC (grade 1 and stage 1). **(A)** Slightly high signal intensity on fat-saturated T2WI. **(B)** The lesion shows enhancement on solid part of ccRCC. **(C)** Pathological analysis confirmed ccRCC (grade 1). **(D)** D map. **(E)** D* map. **(F)** f map. **(G)** MD map. **(H)** KA map. **(I)** MK map. The necrotic areas in the center of the ccRCC. ccRCC, clear cell renal cell carcinoma; T2WI, T2-weighted imaging; MD, mean diffusivity; KA, kurtosis anisotropy; MK, mean kurtosis.

**Figure 3:**
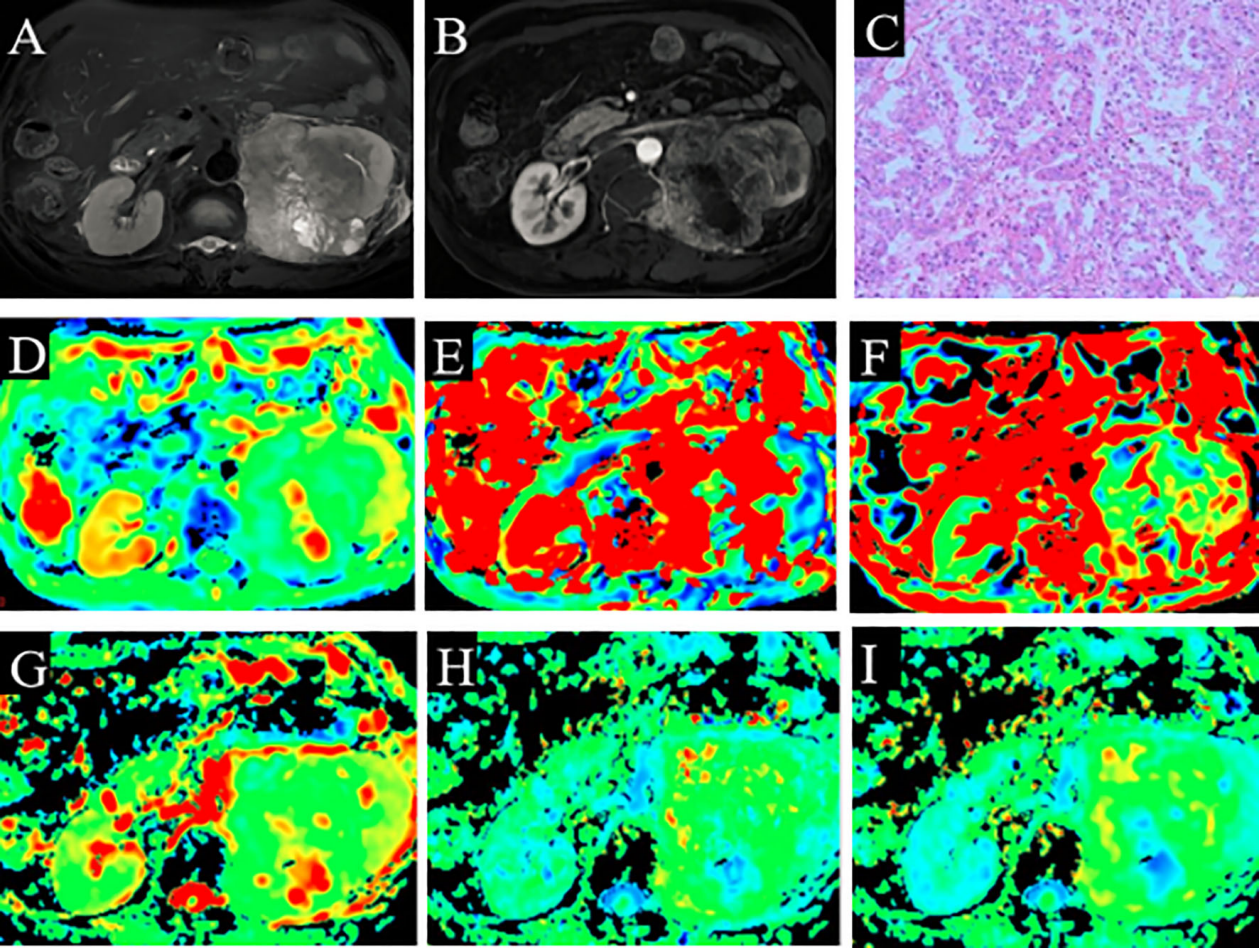
A 66-year-old man with ccRCC (grade 3 and stage 3). **(A)** High signal intensity on fat-saturated T2WI. **(B)** Solid part shows enhancement. **(C)** Pathological analysis confirmed ccRCC (grade 3). **(D)** D map. **(E)** D* map. **(F)** f map. **(G)** MD map. **(H)** KA map. **(I)** MK map. ccRCC, clear cell renal cell carcinoma; T2WI, T2-weighted imaging; MD, mean diffusivity; KA, kurtosis anisotropy; MK, mean kurtosis.

Among the 65 cases of HCC, 39 were low-grade and 26 high-grade patients, and 45 were low clinical stage and 20 were high clinical stage patients. Detailed patient pathological grades and clinical stages are summarized in **Table 1**. **Figures 2** and **3** show the features of low-grade and low-stage ccRCC and high-grade and high-stage on T2-weighted images: D, D*, f, MD, KA, and MK maps. The agreements of IVIM and DKI parameters between two observers were perfect by high ICC for D(0.873), D*(0.838), f(0.786), MD(0.861), KA(0.860), and MK(0.885).

**Table 1:** Pathological grades and clinical stages.

Grade	Stage	Clinical stages			
		Stage 1	Stage 2	Stage 3	Stage 4
WHO/ISUP	G1	13	/	/	/
	G2	21	4	1	/
	G3	7	/	17	1
	G4	/	/	1	/

WHO/ISUP, World Health Organization/International Society of Urological Pathology.

### Comparison of IVIM and DKI parameters between low- and high-grade ccRCC

The D and MD values were higher in low-grade than high-grade ccRCC (*p* = 0.000 and 0.000, respectively), and KA and MK values were lower in low-grade than high-grade ccRCC (*p* = 0.000 and 0.000, respectively). However, the D* and f values had no significant difference in low- and high-grade ccRCC (*p* = 0.185 and 0.088). **Table 2** shows the differences in IVIM and DKI parameters between the low- and high-grade ccRCCs.

**Table 2:** The differences in the IVIM and DKI parameters between the low- and high-grade ccRCC.

	Low-grade	High-grade	t	*p*
D (×10^−3^)	1.538 ± 0.429	1.080 ± 0.300	5.068	0.000
D*	0.077 ± 0.063	0.057 ± 0.030	/	0.185
F	0.286 ± 0.112	0.240 ± 0.121	/	0.088
MD	2.422 ± 0.530	1.834 ± 0.598	4.544	0.000
KA	0.773 ± 0.294	1.067 ± 0.315	−3.795	0.000
MK	0.589 ± 0.258	0.854 ± 0.288	3.791	0.000

IVIM, intravoxel incoherent motion; DKI, diffusion kurtosis imaging; ccRCC, clear cell renal cell carcinoma; MD, mean diffusivity; KA, kurtosis anisotropy; MK, mean kurtosis. D, corresponding diffusion coefficient; D*, pseudo-diffusion coefficient; f, perfusion fraction.

### Comparison of IVIM and DKI parameters between low- and high-stage ccRCC

The D, D*, f, and MD values were higher in low-stage than high-stage ccRCC (*p* = 0.000, 0.005, 0.007, and 0.000, respectively), and the KA and MK values were lower in low-stage than high-stage ccRCC (*p* = 0.000 and 0.000, respectively). **Table 3** shows the differences in the IVIM and DKI parameters between the low- and high-stage ccRCC.

**Table 3:** The differences in the IVIM and DKI parameters between the low- and high-stage ccRCC.

	Low-stage	High-stage	t	*p*
D (×10^−3^)	1.503 ± 0.427	1.021 ± 0.262	4.667	0.000
D*	0.082 ± 0.060	0.040 ± 0.031	/	0.005
f	0.294 ± 0.112	0.213 ± 0.112	/	0.007
MD	2.383 ± 0.515	1.789 ± 0.507	4.334	0.000
KA	0.779 ± 0.292	1.116 ± 0.294	−4.270	0.000
MK	0.603 ± 0.264	0.895 ± 0.268	−4.077	0.000

IVIM, intravoxel incoherent motion; DKI, diffusion kurtosis imaging; ccRCC, clear cell renal cell carcinoma; MD, mean diffusivity; KA, kurtosis anisotropy; MK, mean kurtosis. D, corresponding diffusion coefficient; D*, pseudo-diffusion coefficient; f, perfusion fraction.

### Correlation analyses of IVIM and DKI parameters with histologic grade and clinical stage of ccRCC

The D, D*, and MD values were negatively correlated with the histologic grades (r = −0.524, −0.258, and −0.561, *p* = 0.000, 0.038, and 0.000, respectively) and clinical stages (r = −0.470, −0.413, and −0.516, *p* = 0.000, 0.001, and 0.000, respectively). In contrast, the KA and MK values were positively correlated with the histologic grades (r = 0.504 and 0.542, *p* = 0.000 and 0.000, respectively) and clinical stages (r = 0.478 and 0.421, *p* = 0.000 and 0.000, respectively). The f value was also negatively correlated with the ccRCC clinical stage (r = −0.326, *p* = 0.008). **Tables 4** and **5** show the correlations of IVIM and DKI parameters with the grade and stage of ccRCC, respectively.

**Table 4:** The correlations between IVIM, DKI parameters, and grade of ccRCC.

	IVIM			DKI		
	D	D*	f	MD	KA	MK
r	−0.524	−0.258	−0.211	−0.561	0.504	0.542
*p*	0.000	0.038	0.092	0.000	0.000	0.000

IVIM, intravoxel incoherent motion; DKI, diffusion kurtosis imaging; ccRCC, clear cell renal cell carcinoma; MD, mean diffusivity; KA, kurtosis anisotropy; MK, mean kurtosis. D, corresponding diffusion coefficient; D*, pseudo-diffusion coefficient; f, perfusion fraction.

**Table 5:** The correlations between IVIM, DKI parameters, and stage of ccRCC.

	IVIM			DKI		
	D	D*	f	MD	KA	MK
r	−0.470	−0.413	−0.326	−0.516	0.478	0.421
*p*	0.000	0.001	0.008	0.000	0.000	0.000

IVIM, intravoxel incoherent motion; DKI, diffusion kurtosis imaging; ccRCC, clear cell renal cell carcinoma; MD, mean diffusivity; KA, kurtosis anisotropy; MK, mean kurtosis. D, corresponding diffusion coefficient; D*, pseudo-diffusion coefficient; f, perfusion fraction.

### ROC curve analysis in differentiating histologic grade and clinical stage of ccRCC

**Figure 4:**
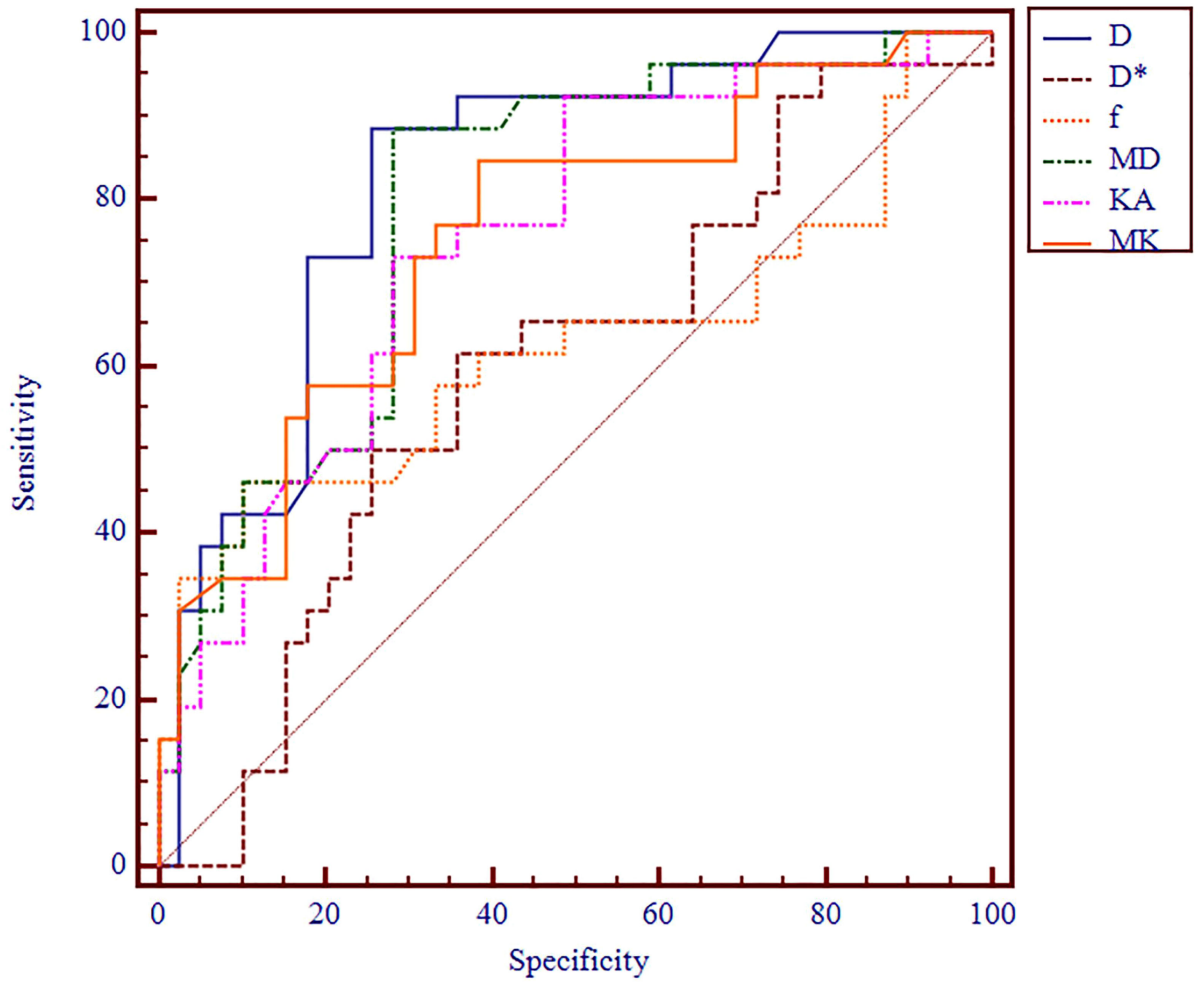
ROC analysis of IVIM and DKI parameters in differentiating histologic grade. ROC, receiver operating characteristic; IVIM, intravoxel incoherent motion; DKI, diffusion kurtosis imaging.

**Figure 5:**
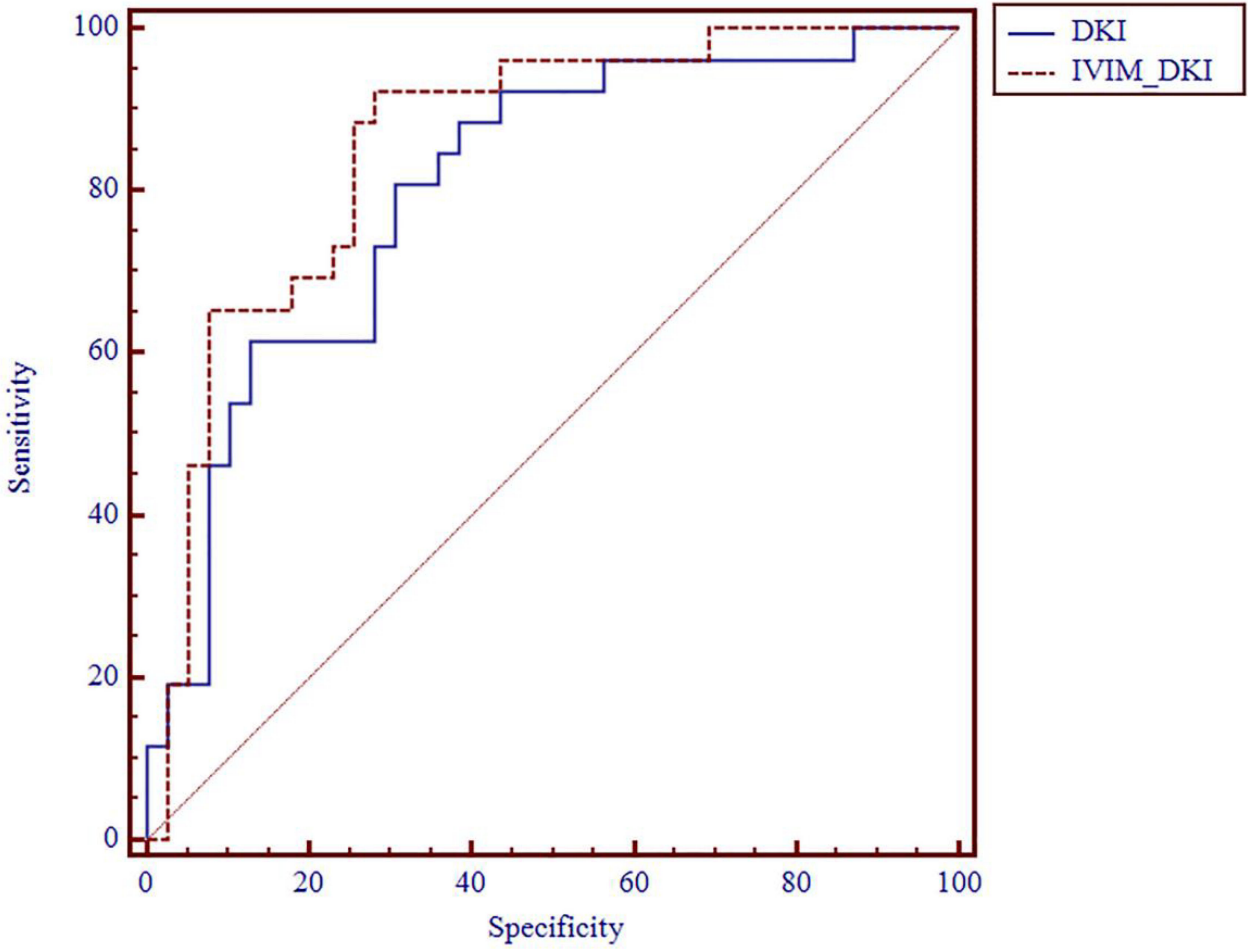
ROC analysis of DKI and IVIM+DKI in differentiating histologic grade. ROC, receiver operating characteristic; DKI, diffusion kurtosis imaging; IVIM, intravoxel incoherent motion.

**Figure 6:**
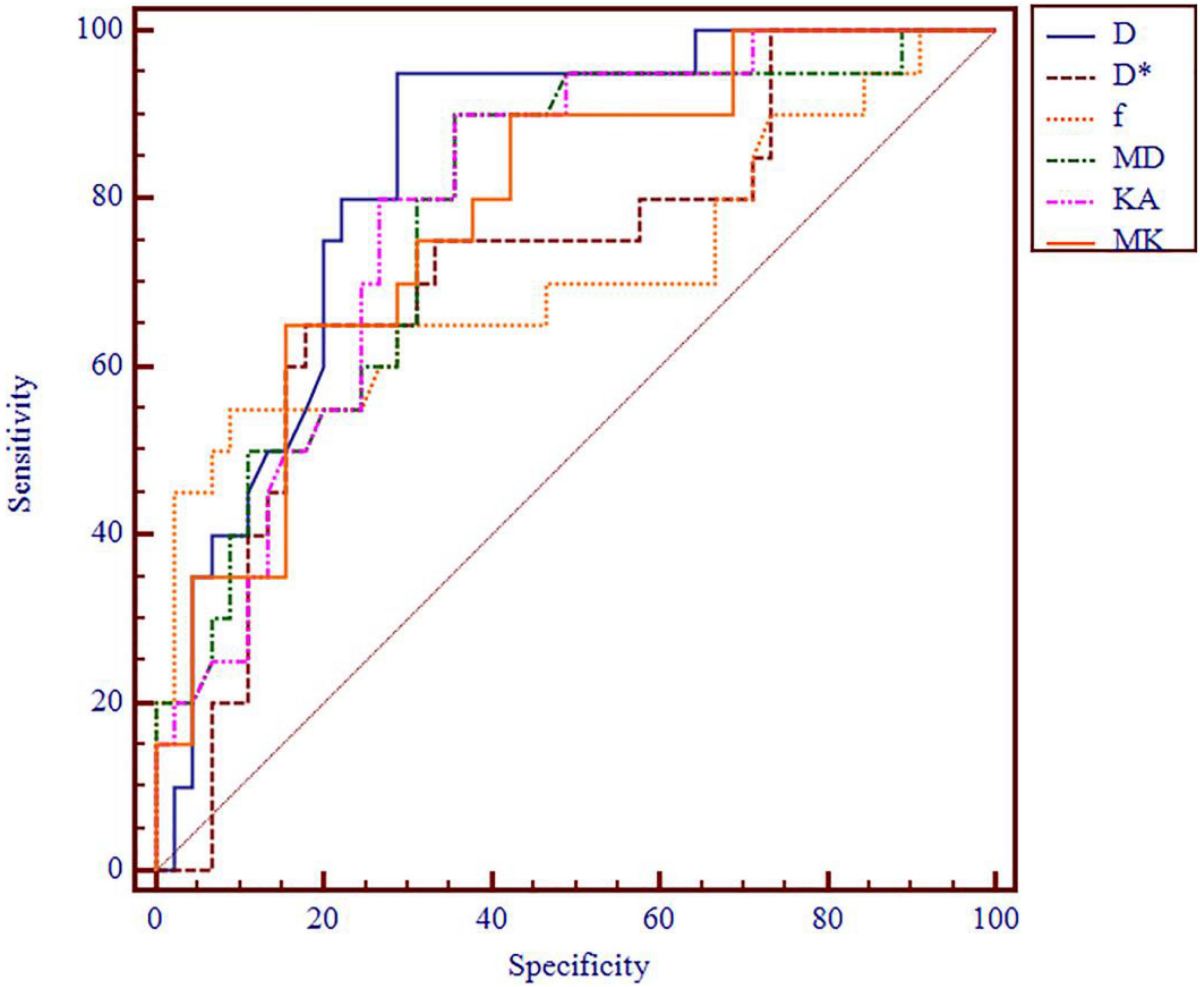
ROC analysis of IVIM and DKI parameters in differentiating clinical stage. ROC, receiver operating characteristic; IVIM, intravoxel incoherent motion; DKI, diffusion kurtosis imaging.

**Figure 7:**
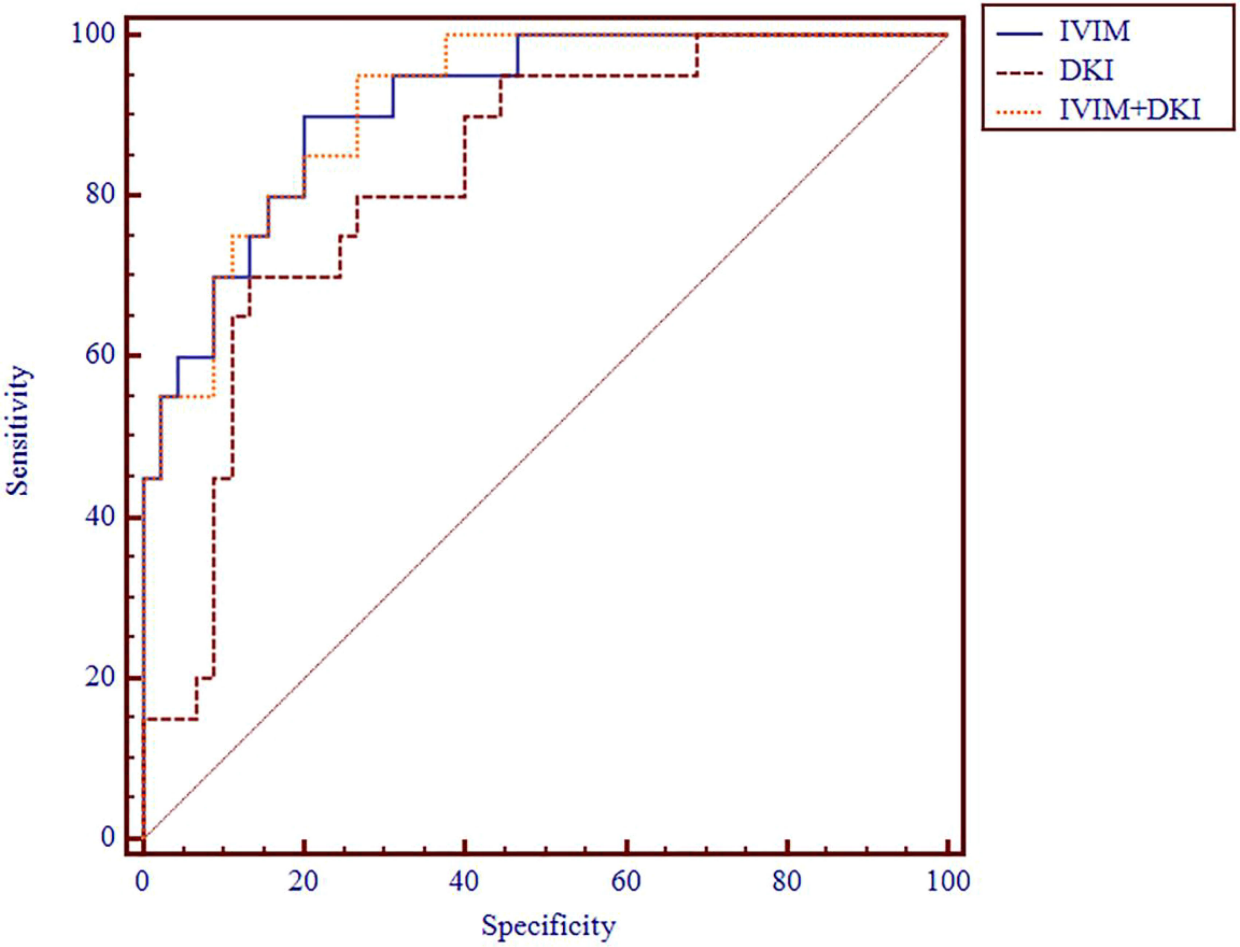
ROC analysis of IVIM, DKI, and IVIM+DKI in differentiating clinical stage. ROC, receiver operating characteristic; IVIM, intravoxel incoherent motion; DKI, diffusion kurtosis imaging.

The AUC values of D, D*, f, MD, KA, and MK values were 0.825, 0.598, 0.626, 0.792, 0.750, and 0.754, respectively, in grading ccRCC and 0.837, 0.719, 0.710, 0.787, 0.796, and 0.784, respectively, in staging ccRCC. The D, MD, KA, and MK values were significant predictors in differentiating low from high grade and stage of ccRCC. Marginally, the model incorporating IVIM and DKI parameters exhibited improved diagnostic capabilities. The AUC values of DKI and IVIM+DKI values were 0.803 and 0.857, respectively, in grading ccRCC and 0.823 and 0.916, respectively, in staging ccRCC, and the AUC of IVIM was 0.913 in staging ccRCC. The AUC values, cutoff values, sensitivity, and specificity in differentiating tumor stage and histologic grade of ccRCC are shown in **Tables 6** and **7** and **Figures 4**–**7**.

**Table 6:** Diagnostic value of IVIM and DKI parameters in differentiating low- and high-grade of ccRCC.

	AUC (area = 0.5)	Cutoff	Sensitivity	Specificity
D	0.825 (*p* < 0.0001)	≤0.0014	88.5	74.4
D*	0.598 (*p* = 0.1798)	≤0.061	61.5	64.1
f	0.626 (*p* = 0.1082)	≤0.173	46.2	89.7
MD	0.792 (*p* < 0.0001)	≤2.28	88.5	71.8
KA	0.750 (*p* < 0.0001)	>0.895	73.1	71.8
MK	0.754 (*p* < 0.0001)	>0.584	84.6	61.5
DKI(MD+KA+MK)	0.803 (*p* < 0.0001)	/	88.5	61.5
IVIM(D)+DKI	0.857 (*p* < 0.0001)	/	92.3	71.8

IVIM, intravoxel incoherent motion; DKI, diffusion kurtosis imaging; ccRCC, clear cell renal cell carcinoma; AUC, area under the curve; MD, mean diffusivity; KA, kurtosis anisotropy; MK, mean kurtosis. D, corresponding diffusion coefficient; D*, pseudo-diffusion coefficient; f, perfusion fraction.

**Table 7:** Diagnostic value of IVIM and DKI parameters in differentiating low- and high-stage ccRCC.

	AUC (area = 0.5)	Cutoff	Sensitivity	Specificity
D	0.837 (*p* < 0.0001)	≤0.0013	95.0	71.1
D*	0.719 (*p* = 0.0020)	≤0.0325	65.0	82.2
f	0.710 (*p* = 0.0086)	≤0.166	55.0	91.1
MD	0.787 (*p* < 0.0001)	≤2.28	90.0	64.4
KA	0.796 (*p* < 0.0001)	>0.791	90.0	64.4
MK	0.784 (*p* < 0.0001)	>0.841	65.0	84.4
IVIM(D+D*+f)	0.913 (*p* < 0.0001)	/	90.0	80.0
DKI(MD+KA+MK)	0.823 (*p* < 0.0001)	/	70.0	86.7
IVIM+DKI	0.916 (*p* < 0.0001)	/	95.0	73.3

IVIM, intravoxel incoherent motion; DKI, diffusion kurtosis imaging; ccRCC, clear cell renal cell carcinoma; MD, mean diffusivity; KA, kurtosis anisotropy; MK, mean kurtosis. D, corresponding diffusion coefficient; D*, pseudo-diffusion coefficient; f, perfusion fraction.

## Discussion

This study explored the value of IVIM and DKI in evaluating tumor grades and T stages in ccRCC patients. The results showed that IVIM and DKI parameters are of great value in differentiating low and high grades and stages, and the D, MD, KA, and MK values have better diagnostic values in grades and stages. In addition, the f values also helped to assess clinical stages. Meanwhile, the diagnostic performance saw a minor enhancement when IVIM and DKI parameters were combined.

IVIM provides a unique view of tissue perfusion without using exogenous contrast agents. The D values of low-grade ccRCC were higher those of than high-grade ccRCC. The degree of tumor differentiation is closely related to its aggressiveness. The IVIM parameters of low-stage ccRCC were higher than those of high-stage ccRCC. The D values represent the water molecule diffusion, which indicates the change in the microenvironment (32). The solid tumor components of RCC have greater cellular density and collagenous interstitial stroma, close arrangement, and small extracellular space, which reduce water diffusion velocity. The D values of low-grade ccRCC were significantly higher than those of high-grade ccRCC (13, 33, 34). Histologic grade and clinical stage were significantly negatively correlated with the D values, and poorer differentiation in histologic grade and clinical stage resulted in lower D values. The reduction in D values can be attributed to the hindrance of water motion due to the increased viscosity of the tumor tissue. Poorer tumor differentiation led to faster proliferation, resulting in increases in the number of tumor cells and the tumor density and a decrease in intercellular substance. The diffusion of water molecules within the tumor tissue was more constrained, which was reflected by significant decreases in D values (35). Yang L et al. (14) explored the value of IVIM in evaluating tumor T stages in locally advanced rectal cancer patients; the D value was significantly distinguished in the diagnosis of staging, and these findings are consistent with ours. Moreover, previous studies have proven negative correlations of D values with the histologic grade in RCC and that D values can be used for histologic grading of RCC (36–39).

The D* and f values are the perfusion parameters, which could be used to analyze the vascularity of the tissue. The D* value is defined as the average blood flow and mainly reflects the capillary blood velocity, and the f value measures the microvascular volume fraction (32). Zhou Y et al. investigated the IVIM of 40 HCC patients and found that the D* and f values did not significantly correlate with the histologic grade (40), being consistent with our study. Huang YC et al. (41) showed that IVIM parameters are negatively correlated with stages of esophageal squamous cell carcinoma, and microvascular volume fraction helps detect and stage esophageal squamous cell carcinoma. As the tumor stage progresses, the central microvessel density decreases while its volume expands. This results in an insufficient central blood supply to the ccRCC, leading to increased micronecrosis (42). The D* and f values of low-grade ccRCC were not statistically significant. The main reasons include 1) the relatively small sample size of ccRCC in the high-grade group; 2) the f value correlated well with the enhancement degree of renal lesions, and partial low-stage ccRCC displays as cystic mass showing slight enhancement, leading the f value of low- and high-grade ccRCC to have no statistical significance (17). In our study, the diagnostic efficiency of the D* and f values was lower than that of the D value, and the AUC of the D value was higher than that of the D* and f values. The limited importance of the D* and f values in this study was explained previously by its high uncertainty and poor reproducibility (43). Additionally, the relatively small sample size might affect the results.

DKI reflects tissue complexity by using higher b values. The signal intensity largely depends on the b values applied, which could identify tumor and necrotic tissue (44). However, a higher b value reduces the signal-to-noise ratio. In our research, the b values used in DKI were 500 and 1,000 s/mm^2^ with 15 diffusion directions per b value. Previous studies have shown that b values of 500 and 1,000 s/mm^2^ are acceptable for kidney tumors, and DKI could provide additional information for revealing the renal microstructure and function (21, 45). Some previous works have confirmed that DKI can effectively distinguish high- from low-grade RCC; this is consistent with the results of this study (22–24). Compared with the results of previous studies, we systematically analyzed the value of DKI in the differentiation and grading of ccRCC, and histologic grade was classified according to the criteria of the WHO/ISUP grade; related research shows that the WHO/ISUP grade system has been considered to be easier to apply to clinical management and superior to the Fuhrman grading system (46). The MD value is corrected by non-Gaussian bias and could give insights into the structural connectivity of tissues, potentially providing useful information on the pathophysiology of diseases. Yang L (14) et al. explored the DKI parameters of the high stage and found that they were lower than those of the low stage in locally advanced rectal cancer patients; in particular, the MD values also yielded comparable overall diagnostic abilities in differentiating the low-stage from high-stage patients. These findings are consistent with ours. Moreover, the MK value identifies deviations of diffusion from Gaussian motions. The changes in organizational structure can affect both MK and KA values (47). Thus, MK and KA are not completely independent, although both indicators can be used to test for different aspects of diffusion. Zhu Q et al. (21) found that papillary RCC has higher MK and KA values as compared with ccRCC due to its aggressiveness and histological heterogeneity. High-grade and stage ccRCC are more aggressive and histologically heterogeneous as compared to low-grade and stage ccRCC and increased the MK and KA values. Moreover, the KA value itself is important in predicting histologic grades and clinical stages of ccRCC.

In our study, we found that the D, MD, KA, and MK values were significantly different in evaluating the histologic grades and clinical stages of ccRCC, whereas the D* and f values were useful only in staging diagnosis Therefore, in the IVIM model, we recommend to combine only the valuable D, D*, and f values for clinical stages. In the DKI model, we recommend combining the valuable MD, KA, and MK values for both histologic grades and clinical stages. Previous research demonstrated that the combined parameters with a *p* < 0.05 from the statistical analysis had the best diagnostic ability (30, 31). Subsequently, we integrated the valuable parameters from both IVIM and DKI models for a more comprehensive evaluation of the histologic grades and clinical stages of ccRCC. This primarily includes the D value from the IVIM model and MD, KA, and MK values from the DKI model for evaluating histologic grades. For clinical staging, we recommend a combination of D, D*, and f parameters from the IVIM model and MD, KA, and MK parameters from the DKI model. Previous research has demonstrated that combining DKI with chemical exchange saturation transfer sequences is more effective than using a single sequence for grading and staging ccRCC (21). In our study, by combining the IVIM and DKI parameters, the resultant model showed a higher AUC than that obtained with a single sequence or parameter.

The limitations of our study include the relatively small sample, which might result in bias in the comparative evaluations. All ccRCC patients received radical or partial nephrectomy treatment. Since all the ccRCC patients in our study underwent either radical or partial nephrectomy, the number of patients with high-grade and advanced-stage ccRCC was relatively low. Further analyses with a larger cohort of ccRCC patients are necessary to study the correlations among different stages and grades.

In conclusion, compared to other relevant research, our study highlights the potential of both IVIM and DKI techniques in systematically characterizing and distinguishing between ccRCC grades and stages, presenting a more effective tool for accurate diagnosis. The combined utilization of the IVIM and DKI models offers enhanced diagnostic accuracy and efficiency. This combination provides valuable insights for clinical decision-making concerning surgical plans, including the choice between partial and radical nephrectomy, and informs patient management during follow-up. Moving forward, we will focus on expanding the sample size, with particular emphasis on including a greater number of cases with high-grade and advanced-stage ccRCC, in order to further validate the correlations with histologic grades and clinical stages.

## Data availability statement

The original contributions presented in the study are included in the article/supplementary material. Further inquiries can be directed to the corresponding author.

## Ethics statement

The studies involving humans were approved by Qilu Hospital of Shandong University ethics committee. The studies were conducted in accordance with the local legislation and institutional requirements. The participants provided their written informed consent to participate in this study. Written informed consent was obtained from the individual(s) for the publication of any potentially identifiable images or data included in this article.

## Author contributions

DY guaranteed the study conception and design. QC carried out the analysis and interpretation of data. AR and XX carried out acquisition of data. QC, ZM, and XF prepared the drafting of manuscript. DY, WD, and DP: critical revision the manuscript. DP for the English revision. All authors contributed to the article and approved the submitted version.
